# Dental Caries

**Published:** 1898-08

**Authors:** Dessie B. Robertson


### Dental Caries.

BY DESSIE B. ROBERTSON, D.D.S.

A Thesis for the Degree of Doctor of Dental Science. University of Michigan, Dental Department, January 30, 1898.

The term, dental caries, as ordinarily used, is a misnomer, being originally used to indicate as a general term, before any accurate knowledge of their nature had been acquired, *several morbid* conditions of the teeth. Particularly was it used in reference to mortifications or ulcerations of bone and was applied to the teeth under the mistaken idea that it, too, was the result of inflamatory action. Now, after the lapse of years, with the wider knowledge which scientific research has brought us, we still continue to use the term, preferring rather to modify its definition than to change the word.

Caries of the teeth, as now understood, consists of a chemical disintegration of the elements of the teeth, molecule by molecule, beginning on the surface in pits or grooves, or other depressions which may offer lodgment for particles of food and which are protected from the washing of saliva or the friction of mastication, and spreading from thence inwards destroying in its progress the structure of the tooth.

As far back in the world's records as history or tradition enable us to go, this disease is found to have prevailed, although it seems probable that it was much less frequent in the earlier ages than at the present time.

The legends of the Egyptians and Hebrews point to its existence and the numerous methods used to alleviate it, such as cauterizing the temples with fire.

Cicero mentions the invention by Esculapius of an instrument for the extraction of carious teeth, while the Hippocratic books contain documents still more precise and authentic, which relate to this malady. Celsus offers a series of prescriptions for use on carious teeth; mixtures of resins or aromatic substances, pepper, opium, and he also advises general treatment, diet, purgatives.

From the Roman epoch to the Renaissance, during which period all scientific knowledge was wrapped in darkness, nothing worthy of note on this topic appears.

At the commencement of the sixteenth century the anatomists began to understand something about the physiology of the teeth and their part in the human economy and following the perfection of the microscope, the constituents and minute structure of these organs being demonstrated, various theories in regard to their pathological conditions began to be formulated.

Let us consider some of the beliefs on this subject, which have from then until now prevailed.

In the first place, it has been generally agreed that, whatever might be the exciting cause of dental caries, there exists also certain conditions which render teeth more prone to disease, divesting them, so to speak, of their original or normal power of resisting the exciting cause and this condition is referred to as the predisposing causes.

Important among these is the structure of the tooth, as in the case of an imperfect enamel where the agent of decay has much better opportunity for action than where a firm, well developed enamel resists its penetration. Not less important are the pits and fissures offering lodgment for food particles, especially in places where the enamel growth not having kept pace with the dentine the latter may be uncovered.

The manner of contact of the proximal surfaces also has much significance. In the triangular spaces often found between the gum and the bicuspids and molars, food particles constantly lodge and fermentation and acid formation continually occur. Indeed, malposition of teeth of whatever nature aids in producing disposition toward disease. Hereditary tendencies cannot be overlooked, as it is well known that a predisposition to badly developed, irregular teeth may be transmitted from parent to child.

These, however, are only secondary factors in the role of decay production.

What, then, *is* the primary cause? This is the question which for more than two hundred years has been occupying the minds of the investigators of different periods, and many are the theories which have been advanced in explanation.

Among the earlier views were those of Bell, in 1787; Serre, in 1788, and Kappis, in 1794, who considered the juices carried to the teeth as holding an important part in causing decay.

Bourdet (1757) declared that when the juices contained in the vessels of the teeth are too thick they stagnate, decay and soon attack the teeth.

John Hunter, in 1778, a careful observer and a deep thinker, wrote upon this subject. A theory had been advanced that decay of the teeth was the result of inflammation, and while he was an advocate of the idea and regarded it as much the same as bone necrosis or mortification of the soft tissues, still he expressed himself as certain that much more remained to be explained, as teeth were not subject to putrefaction after death and there must be some operation going on in life to produce a change in the diseased spot. He also casually remarked the fact of decay beginning at the surface and progressing inward, which to us now seems quite sufficient to have shown him the falacy of his belief in the inflammation theory. The inability of the tooth to repair damage resulting from inflammation or change of temperature was supposed to lead to decomposition of the dead part and formation of a cavity. About 1830 arguments began to be presented against this theory. Harris, Regnard, and many others, by experiments and observation showed it to be illogical.

It had been remarked that artificial teeth made from bone or ivory, or human teeth mounted as substitutes were likely to de cay in the same way as the natural organs, and this could not of course be accounted for by inflammation or any vital process relating to the tooth itself.

The fact that silk threads, or metal clasps fastening artificial teeth were shown to afford lodgment for the fluids of the mouth, and particles of food to become an active cause of dental caries now served to direct the attention of a number of intelligent dentists in a new direction.

Robertson, of England, published his decision which was that caries was the result of chemical decomposition, and that the destruction of the tooth was accomplished by means of acids formed in the mouth and by decomposition of food particles lodged about the teeth.

This theory, denying as it did, not only the influences of inflammation and the vital action of the tooth itself, but also being in contradiction to the then prevalent belief that caries began in the internal part advancing to the surface, met at first with great opposition.

Desirabode, of France, criticising it most severely, declared that caries usually began in the dentine and that it might be deeply affected while the enamel remained entire; that third molars often came from the alveoli deeply decayed, and that at the time of eruption it was impossible they had ever been submitted to the action of any kind of acid, and that if it was an acid which caused decay, then decay would be general, the entire denture being affected.

The failure to grasp the idea intended was perhaps at that time quite natural. Very little of fermentation or the chemical theory in regard to it was understood and nothing of yeast fermentation, so that, while after a time many of the profession began to adopt it, the localization of the development of an acid was not emphasized and it was debated how in a habitually alkaline substance, such as the saliva was known to be, decay by acid could be produced.

But after the first surprise and disapproval had subsided the theory gained in favor, and until very recently has remained with but little modification, the accepted belief of a very large number of distinguished odontologists.

Tomes expressed his opinion in the following terms: "Caries is the effect of external causes in which the so-called vital forces play no part. It is due to the solvent action of acids which have been generated by fermentation going on in the mouth, and when once disintegration is established in some defective point, the accumulation of food and secretion in the oral cavity will intensify the mischief by furnishing new supplies of acids."

Dr. J. Taft also wrote in favor of this theory: "Acid mucous and saliva vitiated secretions, products of decomposition of animal and vegetable matter in the mouth and galvanic action, mineral and vegetable acids, are the chief causes of dental decay."

Magitot in his treatise on dental caries expresses his belief in the acid production of caries and substantiates his opinion by many experiments. He, too, by observation of decay in artificial dentures of bone or ivory disproves the vital theory and affirmed that decay is the result of an external cause.

By treating a large number of extracted teeth with substances which he thought might be present in the buccal cavity in quantities sufficient to produce a marked action, he succeeded in decalcifying the part acted upon and producing a cavity which apparently exhibited all the characteristics of caries.

Experiments were made with solutions of sugar, one part of sugar being used to three parts of water. Perfectly sound teeth were placed in this, a part of these teeth being protected by a coating of wax except at one single point where the enamel was left exposed. After two years the fluid was found to be acid, the teeth not protected were decalcified, while at the point where the wax was wanting a cavity was found.

In another experiment creasote was added to *prevent* the *fermentation*, and at the end of two years the fluid was unclouded and the teeth sound. Another case was reported where the sugar and water having been brought to the boiling point, the mixture with the teeth enclosed was sealed hermetically while hot, the teeth after two years were found to have undergone no change, not even a diminution in weight.

Solutions of various organic acids were also applied with the following results:

Lactic acid 1 part to 100 decalcified.

Butyric acid 1 part to 100 decalcified.

Acetic acid 1 part to 100 did not affect the enamel, acting only on the dentine.

These substances were allowed to act on the teeth for at least two years.

Dr. Magitot's work upon the subject can be described even now as nothing less than elaborate and scientific in every respect. Yet this criticism has been offered, that while he demonstrated that fermentation and acid production, decalcified teeth, he did not put in the foreground with sufficient force the fact that these agents must be formed in contact with the identical point at which caries is manifest.

Again, it will be shown later that the decalcification which he succeeded in producing by means of acids is not exactly identical with the natural decay of a carious tooth.

The mineral acid theory of Dr. George Watt attracted attention both from its novelty and ingenuity. He believed that while organic acids might have their part in producing dental caries the inorganic acids, sulphuric, nitrous, and hydrochloric, were after all the most active agents.

Nitric acid, he claimed, was found in the mouth from decomposition of organic nitrogenous bodies and combinations then being affected between the nitrogen, hydrogen and oxygen.

From the fact that particles of nitrogenous food are often allowed to remain between and around the teeth, he argued that at these points nitric acid was formed and attributed the white form of decay to this agent. Black decay he considered to be the result of sulphuric acid. Sulphur being a decomposition product from albuminous food and combining with both oxygen and hydrogen at the point of attack. That black decay is more slow and acts more feebly he considers due to the fact of its removing only the water, on account of its great affinity for it and leaving a carbonized residue.

Hydro-chloric acid may arise as a product of galvanic action or decomposition of certain chlorides in the food. It was supposed to unite with the carbonate of lime in the tooth substance, forming calcium chloride, which dissolved in the saliva leaving only the organic portion of the tooth and giving rise to soft, pulpy form of caries.

Another most original theory was advanced by Bridgeman, who presented it in an essay on dental caries, which won a prize offered by the Ondontological Society of Great Britian. He considered the mouth as a galvanic battery, the crowns of the teeth being electro positive, the tissue surrounding electro negative. When the fluids of the mouth became abnormal the crown of the tooth yielded to its lime salts, setting free the acids with which they were combined, this being followed by disintegration of the dentine.

Long before any microscopical examination had been made of carious teeth, it was known that in the saliva and in the deposits on the teeth and the margin of the gums small organisms in great number existed. Late in the seventeenth century, Leeuwenhock, a celebrated Dutch scientist, by use of a hand lens was able to detect, in white matter from the teeth, five different kinds of organisms.

Later, various investigators described these forms of life, some designating them as fibres either animal or vegetable, and still later it was decided that they were low forms of plant life, very similar to algae.

After the perfection of the microscope many observers in describing the appearance of carious dentine or enamel, mentioned among the broken down tubules and debris, the presence of these micro-organisms or fungi, but not until eighteen sixty-seven, when Leber and Rottenstein published an account of investigations, had any extended study of bacteria in connection with caries been made. By treating decayed dentine with iodine and acids they obtained a violet coloration of the granular masses in the tubules, and concluded that these masses contained bacteria, a theory which the superior methods of staining has since proved to be a fact. They accepted the acid theory of their predecessors in as much as they considered the micro-organisms themselves to be the acid producers, and held that the micro-organisms did not penetrate the tubuli until by the action of acids, the latter had been rendered less resistant.

This theory, being such a profound departure from former ideas, created much attention, but, from lack of proper methods to demonstrate the facts, it did not receive much support.

In eighteen eighty-one, Milles and Underwood, by means of Dr. Koch's improved staining methods, were able to demonstrate the presence of micro-organisms in the tubules. They made examinations of dentine destroyed by acids alone, under aseptic conditions, and proved that it differed from ordinary decay, in that the tubules in the latter were enlarged, two being broken into one, the intervening matrix being destroyed, while in the former the matrix was uniformly destroyed without any enlargement of the channels and no resemblance in color or consistency was presented. They also showed that while artificial caries, which was an exact counterpart of true caries, could be produced, this could not occur under aseptic conditions and pointed for corroboration of this statement to Magitot's experiments, in which creasote and sealing at boiling temperature prevented fermentation.

Finally they said that the most favorable points for caries were those where a depression or pit in the enamel offered lodgment for bacteria, undisturbed by the action of saliva or mastication.

This theory, while it forms the basis of the now accepted micro-organism theory of caries and while it was a wonderful advance on previous suppositions, still left unexplained many minor points of great importance in regard to the characteristics of these microorganisms, their media and general physiological requirements, and it was left to Dr. Miller, of Berlin, to elaborate and complete up to its present point the chain of evidence which substantiates this belief and renders it the most probable and logical yet advanced.

A brief review of some of the experiments reported by Miller will show how step by step he has confirmed his theory until it now rests on the solid basis of facts. He found that fresh saliva mixed with sugar or starch and kept at incubator temperature becomes acid in from four to five hours, but if put for half an hour in a sterilizer at 100 degrees, it does not sour in a day. From this he concluded that the ferment was rendered inactive by 100 degrees of heat.

Starch previously heated to 150 degrees cooled and mixed with salvia becomes sour, therefore, the ferment could not have existed in the starch.

To discover whether the ferment be organized or unorganized carbolic acid, which in the proportion used, one-half per cent. strong, was known not to injure unorganized ferments, was added, with the result that no acid was found in the solution.

Tubes of sterilized solution of sugar in saliva, infected with carious dentine, became acid, and tubes inoculated with a loop from these tubes likewise show an acid reaction, while the control tube, uninfected with dentine, remained neutral.

The conclusions drawn from these experiments were that in carious dentine or in saliva an organized ferment exists which is capable of producing an acid on carbohydrate media.

By growing under oil or placing the tube in pyrogallic acid or by partial exhausting of the air by an air pump it was found that the quantity of acid was about the same as in the control, so that the micro-organisms in question could grow and produce acid in a limited supply of oxygen, and might therefore exist deep in the dentinal tubules or under fillings, provided other circumstances were favorable.

By cultivation in beef tea or agar a growth was obtained which appeared under the microscope chiefly as diplococci, single or in chains, sometimes as baccilli and in threads. Its character depended greatly, however, on the media, and it was remarked that in beef tea no acid was produced and sections of sterilized dentine in such a solution, infected with cultures of the fungus produced no caries, while a sugar solution under like conditions produced an abundance of acid and the dentine was decalcified.

From its morphology, Miller judged this ferment organism to be similar to the Bacterium Acidi Lactici, and resorted to an analysis of the products of fermentation in order to prove the supposition.

Saliva, containing starch, after standing forty-eight hours, was filtered and heated to 100 degrees to stop fermentation. It was then reduced in volume by heating. Methyl violet added to a few drops produced no effect, showing the acid to be an organic one, and by its non-distilling under heat, it was shown to be a non-volatile acid.

After mixing with ether and distilling an excess of freshly prepared oxide of zinc was added and, after boiling and adding water was put away to crystalize. A few drops on a slide gave characteristic crystals of lactate of zinc. Further quantitative analysis proved this to be a fact, the result obtained from the experiment differing from the formula requirement by less than one per cent.

To sum up the theory, then, he believes caries to be a chemico-parasitical change consisting of two stages. First, decalcification or softening of the tissue, and second, dissolution of the softened residue. The two stages, however, can not be applied to the enamel as in that case decalcification practically signifies destruction. The first stage he believes to be brought about by an acid, the product of fermentation, through the agency of certain micro-organisms acting on charbohydrate substances, which have become lodged in depressions and pits of the teeth and where their acid productions remain in contact with the surface. This acid, which he proved to be lactic acid, combining with the calcium from the calcium phosphate and calcium carbonate of the teeth, forms calcium lactate, which is soluble and thus carried away leaving only the organic substance. Experiments show that of the original lime salts carious dentine retains but one-thirteenth of its original weight.

It is not until the dentine has been decalcified that the bacteria enter the tubules which they fill, and the second stage, the peptonizing of the organic substance, then occurs.

Whether or not among the twenty-two micro-organisms found in the mouth there is one specific bacterium which is *the* cause of caries, has not yet been definitely ascertained; and Miller inclines to the belief that *all* the micro-organisms of the mouth which are capable of causing lactic acid formations may be agents in producing the first stage, and he considers proper conditions being offered, that all, with few exceptions, have this property; while all having the property of peptonizing proteid matter may take part in the second stage, and should any of these fungi possess the power to perform both functions, which power many of them are supposed to have, this one alone may bring about all the changes observed in dental caries.

Miller especially insists, however, on the fact that the dentine must first undergo decalcification before the invasion of bacteria occurs.

Vignal and Galippe working this line, in sympathy with this same theory, in examining eighteen decayed teeth, found four kinds of bacteria present in all. A fifth kind was met with eight times, and a sixth, five times.

They describe these germs as follows:

The first is a short, thick bacillus, not forming chains, grows rapidly in gelatine, forming a white trail, and after three or four days liquifies it.

The second, a long, slender bacillus, slightly constricted in the middle. It forms spreading colonies in gelatine very similar to those of the first kind, with the exception that it spreads farther before liquifying.

The third shows no constriction, forms long chains in liquid media, does not liquify gelatine, merely softening it.

The fourth is a small bacillus which might be mistaken for a coccus. It forms a white trail in the gelatine which turns yellow and then liquifies.

The fifth, which was found but eight times, is a large coccus, and was found only in advanced stages of decay as it was too large to enter the tubuli. This coccus forms white trails in the gelatine, but does not liquify it.

If we take into consideration the physical phenomena of caries we will find that the first signs marking its presence are visible in the enamel, but that the characteristics depend on the surface affected. If it be situated in a fissure a dark spot is usually the first indication, but if the place be smooth, free from indentation, it loses its translucency and becomes opaque, chalky and roughened, a marked discoloration soon after beginning.

The exact cause of this discoloration, varying as it does from very light to brown or black, has been the source of much speculation. Watt, as will be remembered, attributed it to the different mineral acids causing decay. Black explains it on the basis that the coloring matter, such as sulphuretted hydrogen, formed in the mouth, settles in the partly decomposed tissue, and he claims that this is also in harmony with the fact that the more rapid the decay the lighter is the resulting color. Miller thinks this pigmentation due to the presence of chromogenic bacteria, which here, as elsewhere, on organic matter produce their characteristic colors.

The enamel, having been decalcified, is soon mechanically washed away, and an excavation or cavity formed which exposes the dentine. The decay having once penetrated the enamel the destruction of the enamel appears to proceed from the inside, so that an apparently small opening may become so undermined as to open into an extensive cavity. Microscopically examined, the enamel shows broken-down enamel prisms loosened from each other by the acid and mixed with a large number of bacteria.

When the dentine is invaded it becomes softened, cartilaginous, and does not readily fall to pieces, but retains for some time its structural appearance. After a short period, however, it begins to disintegrate, becomes porous and infiltrated with food and masses of bacteria. The progress of the caries seems to be, as much as possible, along the natural openings; that is, along the dentinal tubules toward the pulp, usually in a somewhat cone-shaped form, the apex of which is directed toward the pulp; but there are also many cases where the softening, instead of penetrating to any depth, seems to spread out in all directions between the enamel and the dentine, thus undermining the whole enamel of the crown. The progress in this direction may be traced to the faulty formation of the dentine, such openings being known as inter-globular spaces, or, where an imperfect deposition of lime salts occur, as the granular layer. One peculiarity especially remarked by the best authorities is the transparent zone found adjacent to the boundary of the carious portion of the dentine. This appears in the shape of a zone, its apex toward the pulp. The true cause of this phenomenon has not yet been discovered, although many theories have been advanced.

Tomes and Magitot described it as an effort of nature to impede the progress of the disease and considered it a calcification of the dentinal fibers, but in a later edition of Tomes's works he claims that this zone of transparency has been found in artificially mounted human teeth which have afterwards undergone caries, and so cannot be due to vital action or calcification. Black regards this appearance as the earliest stage of disintegration. Miller thinks no decalcification has been undergone as chemical analysis shows little change in the amount of lime salts, but he considers it a partial or total conversion of fibers into normal dentine.

Microscopical observation of carious dentine shows the bacteria in stained preparations distributed unequally through the softened dentine, large masses being found at the outer margin while towards the inner border areas of decayed dentine are unstained. From this the conclusion is drawn that softening or decalcification of the dentine precedes the invasion by micro-organisms, but as they move more readily along the dentinal tubules than at right angles to them they keep more in pace with the softening towards the pulp than that which extends laterally.

A longitudinal section under low power is described as being crowded with closely packed cells of bacteria, the tubules being enlarged to three or four times their normal size, partly by the neighboring tubules being dislodged, partly by loss of intertubular substance.

It is to be remarked that while during the last century wonderful progress has been made not only in the solving of scientific questions relating to dentistry but also in the mechanical lines such as filling, regulating and furnishing of new and improved appliances, very little advance and it seems very little effort has been made in the direction of prophylactic treatment. To the question of how to prevent or palliate dental caries a brief number of expedients only can be presented, and the general course seems to be to wait until the defect has become apparent and then to remedy it by filling.

This means is very often unsuccessful through the failure of the operator and the structure of the tooth, and it is also a remedy open only to the minority, since the great share of the human race cannot afford the luxury of a first-class dentist.

While it is not probable that the most careful and systematic treatment could completely eradicate caries or not, at least until such methods had been carried through many successive generations, still with our present understanding of its cause it seems as though we ought to be able, to some extent, to mitigate its ravages.

Whether we be an advocate of the chemical or the micro-organism theory, the first step, cleanliness, may be equally applicable. Food material, salivary and mucous deposits and all foreign substances should be removed by means of floss or toothpick or careful manipulation of the brush. Surfaces not exposed to the free action of the saliva or the friction of mastication should be carefully watched and cleansed lest accumulations of food remain there, and all possible mechanical operations be resorted to for thorough purification of the oral cavity. Since the advocacy of the bacteriological theory antiseptic mouth washes of many kinds have been advised, which, following the first course of procedure, should certainly offer good results. All media possible for their retention being mechanically removed and the bacteria themselves being exterminated, or at least inhibited, some degree of asepsis surely should prevail.

But experiment has shown that perfect disinfection of the oral cavity is not, after all, such an easy matter. In the first place the disinfectant used must be mild in character, since powerful agents undoubtedly qualified to destroy the micro-organisms cannot be introduced into the mouth. The time of its retention must necessarily be brief, much less in fact than fluids under ordinary conditions can be left in contact with septic matter.

It is also impossible for the germicidal substance to penetrate all the fissures and inter-proximal spaces, and no matter how carefully mechanical cleansing has preceded its use, there is certain to remain particles of food containing micro-organisms which are not affected.

Miller, in his experiments with antiseptic mouth washes, reports perfect sterilization with mercuric chloride in one-half to three-quarters of a minute, but personal experimentation has given different results.

Miller's general method of procedure was followed. Tubes of beef tea bouillon were used, the first or control tube being infected with saliva before the use of the disinfectant. After this the disinfectant was held and rinsed in the mouth for from ten to twenty seconds and then placed in a sterile Esmark dish. With a small loop (the same as used in making the control) transfers were made from this into large tubes of bouillon at periods of two and one-half, five, ten and fifteen minutes, and the tubes placed at incubator temperature.

After one day the following results were noted:

| DISINFECTANT. | 2½ MIN. | 5 MIN. | 10 MIN. | 15 MIN. |
|---|---|---|---|---|
| $HgCl_2$ 1-2000 | +abundant growth | +abundant | +slight | +slight |
| Mercury soap 1-5000 | +abundant | +abundant | +abundant | +moderate |
| Mercury soap 1 percent | +slight | +slight | +very slight | +very slight |
| Salicylic acid 1-300 | +abundant | +abundant | +abundant | +abundant |
| Benzoic acid 1-300 | +moderate | +moderate | +slight | +very slight |
| Miller's Mouth Wash 1·7 | + | + | +slight | +very slight |
| Sodium Salicylate 1-300 | +abundant | +abundant | + | + |
| Sodium Benzoate 1-300 | + | + | + | + |

| DISINFECTANT. | $2\frac{1}{2}$ MIN. | 5 MIN. | 10 MIN. | 15 MIN. |
|---|---|---|---|---|
| Euthymol, full strength | +slight | +very slight | +very slight | +traces |
| Euthymol, full | +very slight | +very slight | +very faint | +traces |
| Formaline 2 percent | + | +faint | + | |
| Formaline 1 percent | +faint | +faint | +very faint | +very faint |
| Formaceptol, full strength | +faint | +faint | +very faint | +very faint |
| Borolyptol | +faint | +very faint | no growth | +slight growth |
| Listerine, full strength | +slight | + | + | nogrowth |
| Lysol 1 percent | no growth | no growth | +very faint | + very faint |

That these results differed so from Miller's record might be accounted for in several ways. The tubes of bouillon were large, containing about 10 c.c. and a small loop was used, so that very little disinfectant substance was carried into the tube. In his description of his work neither the amount of the bouillon or the size of the loop is mentioned, so that should a large loop have been used in a small amount of bouillon sufficient of the antiseptic substance may have been transferred to inhibit the growth in the tubes.

These experiments, however, are not conclusive in regard to the actual number of germs destroyed by the substance. A tube may be densely crowded with growth as the result of only a very few extremely resistant germs which have existed and multiplied rapidly.

To ascertain the exact numerical decrease in the growth, cultures in agar, in Petri dishes, were made, and in this case the saliva was collected in a sterilized Esmark dish and then filtered through a sterilized glass wool filter into a second sterilized dish. A control was made and poured into a Petri dish and afterwards the saliva and equal parts of the disinfectant to be tested were mixed in a sterile dish. From this mixture, at intervals of two and one-half, five, ten and fifteen minutes, Petri dish cultures were made and set in the incubator for one day when the colonies were counted, and for fear all should not have in that time developed they were returned to the incubator and counted again the second day.

The following shows the result of the same saliva obtained at different times of day. The first was taken from the mouth in the morning, no antiseptic mouth wash having been used for at least twelve hours, the second in the middle of the day six hours after using an antiseptic mouth wash:

Number of colonies in control—1,043.

| | 2½ MIN. | 5 MIN. | 10 MIN. | 15 MIN. |
|---|---|---|---|---|
| $HgCl_2$ 1-2000 | 143 | 81 | 75 | 42 |
| Lysol 1-100 | no growth | 13 | 26 | no growth |
| Listerine, full strength | 4 | 6 | 9 | 3 |
| Borolyptol, full strength | 500 | 480 | 630 | 475 |
| Formaceptol, full strength | 36 | 38 | 34 | 2 |
| Euthymol, full strength | no growth | 3 | no growth | 3 |
| Miller's Mouth Wash 1-7 | no growth | 1 | no growth | 2 |

EXPERIMENT 2.

Control—432 colonies.

| | 2½ MIN. | 5 MIN. | 10 MIN. | 15 MIN. |
|---|---|---|---|---|
| Listerine, full strength | 1 | 2 | 1 | 1 |
| Borolyptol, full strength | 200 | 130 | 140 | 95 |
| Formaceptol, " " | 100 | 5 | 1 | 75 |
| Lysol, 1-100 | 40 | 0 | 0 | 0 |
| Euthymol, full strength | 0 | 0 | 0 | 0 |
| Miller's Mouth Wash, 1-7 | 23 | 4 | 27 | 13 |
| Formaline 2 percent | 150 | 54 | 67 | 36 |
| $HgCl_2$ | 300 | 235 | 228 | 84 |

Seven experiments were made in this way, different saliva being used, in order to determine, if possible, what mouth washes, in the majority of cases, were most effective.

Comparisons of the tabulated results show that in most cases listerine and euthymol, in full strength, lysol 1-100, and Miller's mouth wash, 1-7, brought about the most satisfactory results. The number of the colonies, as shown by the control, being reduced from several hundred thousands to three, four or five, and in some instances no growth appearing.

It must be remembered, however, that the antiseptics in these cases are acting under even more favorable conditions than it would be possible to procure in the mouth. The masses of bacteria, so unfavorable for perfect action, have been broken up by filtering, the antiseptic is throughly mixed with the infected matter and remains in contact with it much longer than when used as a mouth wash.

When we consider that even with all these favoring conditions, after five, ten or fifteen minutes contact with the mouth wash, growths are found in the agar, we cannot but conclude that *perfect* sterilization of the oral cavity is practically impossible.

From the very structure of the teeth and the form of the gum margin there is afforded such ample lodging places for food and the germination of bacteria that, no matter how careful mechanical means may first be applied, all these interstices cannot be penetrated by the antiseptic with sufficient power to destroy all vitality. Consequently we *cannot* say that *perfect sterilization, entire destruction* of all bacterial life has been accomplished.

While we cannot claim that antiseptic mouth washes will produce perfect asepsis, intelligent application will undoubtedly do much to secure this desirable condition. As has been shown, the number of germs may be reduced to a very small percent of their original number, while the growth of those remaining may be inhibited to a marked degree.

The first action of a disinfectant on a germ is to inhibit its growth. This was demonstrated in this series of experiments from the fact that the number of colonies on the Petri dish was much greater after remaining two days in the incubator than on the first day's observation, showing that the presence of the antiseptic had, in some degree, inhibited their growth and produced a slow development.

Besides retarding its growth, inhibition of a germ includes a cessation in reproduction and also in elimination of its characteristic products.

This last fact indicates a practical procedure which should be utilized in efforts to secure sterilization of the mouth. If we can decrease to a large extent the number of bacteria or render the greater part remaining powerless, for a time at least, to produce acid or peptonizing substances, we have, while not doing away with their presence, placed them in a position to do a minimum amount of damage to tooth structure. To attain this desirable end, careful mechanical cleansing, together with frequent and thorough use of proper antiseptics, should be brought to the notice of our patients. Its value and importance cannot be too strongly advocated as one of the most rational measures for overcoming and preventing dental caries. It will also be evident that too much dependence on the value of the mouth wash, as determined by any scientific research, is liable to result unsatisfactorily or perhaps disastrously because of the peculiar conditions of the mouth, and that these disinfectant mouth washes, however powerful, should be looked upon as only one of several important means to an end. Frequent mechanical cleansings are of the greatest benefit, but these two remedies, accompanied by careful and systematic attention in the way of preserving the teeth and inter-proximal spaces intact and in normal relationship, will undoubtedly accomplish more in the way of preventing the development of caries than any other known method of treatment.

